# Cholinergic Submucosal Neurons Display Increased Excitability Following *in Vivo* Cholera Toxin Exposure in Mouse Ileum

**DOI:** 10.3389/fphys.2018.00260

**Published:** 2018-03-21

**Authors:** Candice Fung, Katerina Koussoulas, Petra Unterweger, Andrew M. Allen, Joel C. Bornstein, Jaime P. P. Foong

**Affiliations:** ^1^Department of Physiology, The University of Melbourne, Parkville, VIC, Australia; ^2^Florey Institute of Neuroscience and Mental Health, The University of Melbourne, Parkville, VIC, Australia

**Keywords:** enteric nervous system, enteric circuitry, cholera toxin, diarrhoeal disease, cholinergic neurons

## Abstract

Cholera-induced hypersecretion causes dehydration and death if untreated. Cholera toxin (CT) partly acts via the enteric nervous system (ENS) and induces long-lasting changes to enteric neuronal excitability following initial exposure, but the specific circuitry involved remains unclear. We examined this by first incubating CT or saline (control) in mouse ileal loops *in vivo* for 3.5 h and then assessed neuronal excitability *in vitro* using Ca^2+^ imaging and immunolabeling for the activity-dependent markers cFos and pCREB. Mice from a C57BL6 background, including *Wnt1*-Cre;R26R-*GCaMP3* mice which express the fluorescent Ca^2+^ indicator GCaMP3 in its ENS, were used. Ca^2+^-imaging using this mouse model is a robust, high-throughput method which allowed us to examine the activity of numerous enteric neurons simultaneously and *post-hoc* immunohistochemistry enabled the neurochemical identification of the active neurons. Together, this provided novel insight into the CT-affected circuitry that was previously impossible to attain at such an accelerated pace. Ussing chamber measurements of electrogenic ion secretion showed that CT-treated preparations had higher basal secretion than controls. Recordings of Ca^2+^ activity from the submucous plexus showed that increased numbers of neurons were spontaneously active in CT-incubated tissue (control: 4/149; CT: 32/160; Fisher's exact test, *P* < 0.0001) and that cholinergic neurons were more responsive to electrical (single pulse and train of 20 pulses) or nicotinic (1,1-dimethyl-4-phenylpiperazinium (DMPP; 10 μM) stimulation. Expression of the neuronal activity marker, pCREB, was also increased in the CT-treated submucous plexus neurons. c-Fos expression and spontaneous fast excitatory postsynaptic potentials (EPSPs), recorded by intracellular electrodes, were increased by CT exposure in a small subset of myenteric neurons. However, the effect of CT on the myenteric plexus is less clear as spontaneous Ca^2+^ activity and electrical- or nicotinic-evoked Ca^2+^ responses were reduced. Thus, in a model where CT exposure evokes hypersecretion, we observed sustained activation of cholinergic secretomotor neuron activity in the submucous plexus, pointing to involvement of these neurons in the overall response to CT.

## Introduction

Cholera toxin (CT), produced by *Vibrio cholera*, causes severe diarrhea and cholera remains a major health issue in developing countries. It is well-established that CT induces hypersecretion of water and electrolytes in various animal models *in vivo* (De and Chatterje, 1953; Basu and Pickett, 1969) and *in vitro* (Field et al., 1972; Carey and Cooke, 1986; Burleigh and Borman, 1997). Although CT has been extensively studied using these animal models, the underlying mechanism responsible for its effects remains a matter of contention. Ambiguities have arisen partly due to differences in the types of preparations and methods (*in vivo* vs. *in vitro*) used between studies. For instance, *in vitro* studies using rabbit and human mucosal monolayers indicate that the mucosa alone is sufficient for CT to evoke hypersecretion (Field et al., 1972; Moriarty et al., 1989; Burleigh and Borman, 1997; Burleigh and Banks, 2007). Whereas *in vivo* studies in rats and cats demonstrate the importance of the enteric nervous system (ENS), as specific neural blockers attenuate CT hypersecretion (Cassuto et al., 1982, 1983; Jodal et al., 1993; Sjöqvist et al., 1993; Mourad et al., 1995; Turvill et al., 2000; Kordasti et al., 2006). Furthermore, *in vitro* incubation of CT in the lumen of guinea pig jejunum induces hyperexcitability of specific subtypes of enteric neurons, including secretomotor neurons (Gwynne et al., 2009) and a subset of sensory neurons (Koussoulas et al., 2017). *in vitro* secretion studies in guinea pig implicate both a direct mucosal effect and a neural contribution to CT-evoked hypersecretion (Carey and Cooke, 1986). However, the extent of the contribution from direct CT effects on the mucosa, or indirect effects via the ENS, to the hypersecretion remains unclear. Furthermore, how CT affects the overall neuronal activity within the integrated enteric network remains to be elucidated.

The ENS consists of two ganglionated plexuses: the submucosal and the myenteric plexus situated within the walls of the gastrointestinal tract. The submucosal plexus contains efferent secretomotor neurons and is the main regulator of intestinal secretion, and hence has been the focus of most CT studies. However, CT also alters intestinal motility (Mathias et al., 1977; Koch et al., 1983; Kordasti et al., 2006; Fung et al., 2010; Balasuriya et al., 2016) which is a function mostly ascribed to the myenteric plexus. There is limited evidence that the myenteric plexus may be involved in CT-induced hypersecretion as chemically ablating this plexus in rats can inhibit the secretory response (Jodal et al., 1993). By contrast, in human and guinea pig ileal tissue, CT still induced a secretory response in preparations with the myenteric plexus removed (Carey and Cooke, 1986; Burleigh and Borman, 1997). While the myenteric plexus may not be essential for CT-hypersecretion, the extent of its contribution (if any) to the secretory response is unclear.

Interspecies differences are another confounding factor in the interpretation of CT studies. Findings from *in vivo* rat studies suggest that CT indirectly activates afferent enteric pathways by stimulating serotonin (5-HT) release from enteroendocrine cells (Beubler et al., 1989), which in turn excites intrinsic sensory nerve endings in the mucosa via 5-HT_3_ receptors (Turvill and Farthing, 1997; Kordasti et al., 2006). While it is has been thought that overstimulation of secretomotor pathways by excess 5-HT underlies CT hypersecretion (Lundgren and Jodal, 1997), more recent findings in the guinea-pig suggest that CT acts by increasing the excitability of enteric circuits (Gwynne et al., 2009; Koussoulas et al., 2017). In mice and guinea pigs, cholinergic [choline acetyltransferase (ChAT)-containing] and non-cholinergic [vasoactive intestinal peptide (VIP)-containing] secretomotor neurons are distinct subtypes (Furness, 2000; Mongardi Fantaguzzi et al., 2009). However, some submucosal neurons express both ChAT and VIP in humans (Schneider et al., 2001; Anlauf et al., 2003; Krueger et al., 2016), although acetylcholine (ACh) and VIP may be independently released. Studies performed on human tissues *in vitro* and on rats *in vivo* indicate that CT-induced secretion is predominantly mediated by VIP released by secretomotor neurons, as various VIP antagonists attenuate cholera hypersecretion (Mourad and Nassar, 2000; Banks et al., 2005; Kordasti et al., 2006). Nevertheless, there are considerable discrepancies between these studies, as the effectiveness of specific VIP antagonists in attenuating CT-induced secretion has been reported to be inconsistent. Some *in vitro* studies in guinea pig small intestine and human epithelial cells also implicate ACh (Carey and Cooke, 1986; Banks et al., 2004; Gwynne et al., 2009). While blocking nicotinic receptors *in vivo* attenuates CT-evoked hypersecretion by inhibiting the major form of excitatory transmission within the enteric circuitry, muscarinic antagonists do not have significant effects (Cassuto et al., 1982; Kobayashi et al., 2001; Kordasti et al., 2006). Thus, the relative contributions of cholinergic and non-cholinergic secretomotor neurons to CT-induced hypersecretion remain to be elucidated.

The mouse is a commonly-used model for screening cholera vaccines and therapies (Izzo et al., 2003; Thiagarajah et al., 2004; Rivera et al., 2013; Sawasvirojwong et al., 2013; Chatterjee et al., 2015). However, these studies have focused on CT effects on the mucosal epithelium. In this study, we examined the effects of CT on the ENS in mouse small intestine. A combination of *in vivo* and *in vitro* techniques was used. We have previously reported that the effects of CT persist for hours following initial exposure (Gwynne et al., 2009; Koussoulas et al., 2017). Thus in this study, CT was incubated acutely in an ileal loop *in vivo* (Burrows and Musteikis, 1966; Basu and Pickett, 1969; Sawasvirojwong et al., 2013) aiming to more closely mimic conditions in the whole animal, and then we performed *in vitro* analyses to examine the effects of CT exposure on the enteric circuitry. To elucidate the effects of CT on the integrated enteric network, we incubated CT in live transgenic mice in which enteric neurons and glia express the genetically encoded fluorescent Ca^2+^ indicator GCaMP3 (Danielian et al., 1998; Zariwala et al., 2012). Compared to loading tissues with calcium indicator dyes, these transgenic mice provide advantages of improved signal-to-noise ratio and tissue viability (Boesmans et al., 2013, 2015a), both of which are especially important given our model of *in vivo* CT incubation. Surprisingly, we found that cholinergic, rather than VIP, submucosal neurons displayed a sustained increase in excitability following CT incubation.

## Materials and methods

### Mice

Eight to twelve week old male mice on a C57BL6 background, including *Wnt1*-Cre;R26R-*GCaMP3* mice (Danielian et al., 1998; Zariwala et al., 2012) in which the ENS expresses the Ca^2+^ indicator GCaMP3 were used. *Wnt1*-Cre;R26R-*GCaMP3* mice were generated by mating female homozygous floxed R26R-*GCaMP3* mice with male heterozygous *Wnt1*-Cre mice. All procedures were approved by the University of Melbourne Animal Experimentation Ethics Committee (application ID 1312808).

### *In vivo* incubation of CT in a ligated ileal loop

Mice were removed from food overnight and were anesthetized deeply using isoflurane (1.5–2%) by inhalation until loss of pedal withdrawal reflex. Bupivacaine hydrochloride (0.05%) was administered locally at the site of abdominal incision. A laparotomy was then performed and a 3–4 cm ileal loop was constructed from a region of ileum that was selected because it was as clear of gut contents as possible. The oral and anal ends of the chosen loop were tied off with surgical suture, and the anal end was at least 3 cm proximal to the ileocecal junction. The segment was then injected with 300 μl of either sterile physiological saline (composition in mM: NaCl 118, NaHCO_3_ 25, D-glucose 11, KCl 4.8, CaCl_2_ 2.5, MgSO_4_ 1.2, NaH_2_PO_4_ 1.0) as a control, or CT (12.5 ug/ml; List Biologicals, Campbell, CA, USA) in physiological saline. This concentration of CT is within the range that effectively induced changes in gastrointestinal motility in male mice (Balasuriya et al., 2016), increased the excitability of specific enteric neuronal subtypes in guinea pigs (Gwynne et al., 2009; Koussoulas et al., 2017), and increased secretion in rat and human intestine (Burleigh and Borman, 1997; Kordasti et al., 2006). The abdomen was closed with stitches and the mice were allowed to recover from the anesthesia. During the recovery phase, the animal had access to drinking water. Following 3.5 h incubation post-surgery, the mice were killed by cervical dislocation. The ileal loop was removed from abdomen and flushed clean of the luminal contents prior to *in vitro* analyses. In some experiments, 1–2 cm segments of ileum ~2 cm proximal to the ileal loop, the jejunum, and the proximal colon were also collected to assess potential off-target effects.

### *In vitro* measurement of short-circuit current in ussing chambers

Full thickness preparations in which all intestinal layers were intact were used in Ussing chamber experiments. Each ileal loop provided up to 2 Ussing chamber preparations that were set up as described previously (Foong et al., 2010; Fung et al., 2014). Briefly, each preparation was mounted across an opening [5.5 mm pin circle diameter, 4 mm reservoir opening, CHM8; World Precision Instruments, Inc. (WPI), Sarasota, FL, USA] to divide the two halves of the Ussing-chamber. Short-circuit current (I_SC_) was measured throughout the experiment. Preparations were equilibrated for 30 min prior to collecting data to allow for the basal I_SC_ of the tissue to stabilize and subsequently recorded for 90 min. Data collection and analysis were performed using AcqKnowledge 3.9.0 software (BIOPAC Systems, Inc., SDR Clinical Technology, Middle Cove, NSW, Australia).

### Ca^2+^ imaging and analysis

Ileal loops and ileal segments proximal to the loop from *Wnt1*-Cre;R26R-*GCaMP3* mice were used for Ca^2+^ imaging. The tissue was opened along the mesenteric border and pinned flat with mucosa up in a silicone elastomer-coated dish. The submucosal plexus with the mucosa intact were obtained by microdissection as previously described (Fung et al., 2017). The circular muscle was then removed to obtain myenteric plexus preparations. The resulting submucosal and myenteric plexus preparations were stabilized by stretching the tissue with the plexus facing up over an inox ring and then clamped with an O-ring for imaging (Vanden Berghe et al., 2002). Up to 2 ring preparations of each plexus (submucosal and myenteric) were obtained from each ileal loop. Dissections took no longer than 40 min. Preparations were constantly superfused (1 ml min^−1^) with 95% O_2_: 5% CO_2_-bubbled physiological saline at room temperature throughout the experiment. Ring preparations were imaged through an Olympus 20 × (NA 0.5) water dipping lens on an upright Zeiss Axioskope microscope with a Zeiss AxioCam MRm camera and images (278 × 278) were acquired at 1 Hz.

Preparations were first imaged for spontaneous Ca^2+^ activity prior to stimulation and each ganglion of interest was imaged for 2 min. As ACh acting on nicotinic receptors is the primary means of neurotransmission within the ENS, 1,1-dimethyl-4-phenylpiperazinium (DMPP, nicotinic agonist) is often used to stimulate enteric neurons (Foong et al., 2015). Hence, preparations were stimulated either chemically using DMPP or electrically. DMPP (10 μM) was applied to the preparations by pressure ejection using a spritz pipette positioned adjacent to the ganglion of interest (500 ms duration; 9 psi). Electrical stimuli (single pulse or train of stimuli: 300 μs, 20 pulses, 20 Hz) were applied via a stimulating electrode (50 μm non-insulated tungsten wire) on an internodal strand leading to the ganglion imaged. These stimulation regimes are typically used in the ENS to evoke fast and slow synaptic potentials (Foong et al., 2012). Each stimulation was separated by at least a 5 min interval.

Ca^2+^ imaging analyses were performed using Igor Pro (Wavemetrics, Lake Oswego, Oregon, USA) (Boesmans et al., 2013). The amplitude of each [Ca^2+^]_i_ peak was calculated for each response and was measured as the maximum increase in fluorescence from baseline (ΔFi/F0). [Ca^2+^]_i_ transients were considered when the signal increased above baseline by at least 5 times the intrinsic noise. The frequency of spontaneous [Ca^2+^]_i_ transients was determined by counting the total number of transients within the 2 min recordings and is presented as the average number of transients per minute for each neuron. The total duration of Ca^2+^ imaging experiments lasted up to 4.5 h following dissection of the tissue out of the mice. At least 3 animals were examined for each condition. The size of responding myenteric neurons were measured using ImageJ software (NIH) by manually tracing the outline of the neuronal cell body based on the GCaMP3 signal and then calculating the area (pixels^2^).

After calcium imaging experiments, preparations were fixed in 4% formaldehyde/PBS overnight at 4°C for *post-hoc* immunohistochemistry.

### Electrophysiology: intracellular recording

Tissues were collected in physiological saline containing nicardipine (2.5 μM) and hyoscine (1 μM) (both from Sigma Aldrich, Castle Hill, NSW, Australia) bubbled with 95% O_2_: 5% CO_2_. Myenteric plexus preparations were obtained by microdissection as previously described (Foong et al., 2012). Dissections took no longer than 30 min. As the mucosa was dissected away, CT was absent during recordings. Preparations were superfused with physiological saline and 95% O_2_: 5% CO_2_ at 37°C and allowed to equilibrate for 1 h prior to beginning recording. The total duration of recordings lasted up to 4.5 h.

Myenteric ganglia were viewed at X200 magnification through a long distance objective (LMPlanF1 20x/0.40 ∞/o; Olympus, NSW, Australia) and 10X eyepieces. Using standard intracellular recording techniques (Foong et al., 2012), neurons were impaled with glass microelectrodes (resistance 95–200 MΩ, 1 M KCl, with or without 2% biocytin (Sigma Aldrich) to label recorded cells.

Focal electrical stimulation was applied using a monopolar stimulating electrode (50 μm, tungsten wire) positioned on an internodal strand leading into the ganglion. Single stimuli were applied to evoke fast excitatory postsynaptic potentials (EPSPs). The amplitude (change in mV, from baseline to peak amplitude) of fast EPSPs was quantified. The overall resting membrane (RMP) of the neurons, and spontaneous activity were also examined. The durations of recordings that were used to detect spontaneous fast EPSPs in both control and CT-treated preparations were comparable (Control: 3.7 ± 0.7 min; CT 3.1 ± 1 min; *P* > 0.5).

### Immunohistochemistry

Fixed wholemount preparations, Ca^2+^ imaging preparations, and cryosections were used for immunohistochemical labeling. Wholemount preparations were permeabilized with 1% triton X-100/PBS (ProSciTech, Thuringowa, QLD, Australia) for 30 min at room temperature and washed in PBS (3 × 10 min). Preparations of submucosal plexus and myenteric plexus were incubated with various combinations of primary antisera (Table 1) for 48–72 h at 4°C. Following PBS washes (3 × 10 min), preparations were incubated with secondary antisera (Table 1) for 2 h at room temperature. Preparations labeled with rabbit α pCREB or rabbit α c-Fos were first incubated in biotinylated donkey α rabbit IgG (1:100; Jackson Immuno Labs, West Grove, Pennsylvania, USA) for 2 h at room temperature. These preparations were then washed in PBS (3 × 10 min) and subsequently incubated with streptavidin AF594 (1:200; Molecular Probes, Eugene, Oregon, USA) and secondary antisera (Table 1) for 2 h at room temperature. Ca^2+^ imaging preparations were first incubated with a blocking buffer containing 4% donkey serum (Jackson Immuno Labs) and 0.5% triton X-100 (Sigma Aldrich) in PBS overnight at 4°C. Primary and secondary antisera applied to these preparations were also diluted in blocking buffer. Ca^2+^ imaging preparations were then incubated with various combinations of primary and secondary antisera (Table 1) for 48–72 h at 4°C as described for wholemount preparations. Cryosections (10 μm) were incubated in a goat α CT-B (Table 1) overnight at 4°C. Excess antisera were washed away with 3 × 10 min PBS washes. Sections were then incubated with a secondary antiserum for 3 h at room temperature. Preparations were washed in PBS (3 x 10 min) prior to mounting onto slides using Dakocytomation fluorescence mounting medium (Carpinteria, CA, USA).

**Table 1:** Primary and secondary antibodies used for immunohistochemistry.

**Primary antibodies**	**Host**	**Dilution**	**Source**	**References**
pCREB	Rabbit	1:1,000	Millipore	Chen et al., 2007
c-Fos	Rabbit	1:5,000	Oncogene	Bjerknes and Cheng, 2001
ChAT	Goat	1:500	Chemicon	Foong et al., 2014
VIP	Rabbit	1:1,000	Millipore	Foong et al., 2014
nNOS	Sheep	1:1,000	Gift from P. Emson	Qu et al., 2008
Calretinin	Goat	1:1,000	SWANT	Qu et al., 2008
Hu	Human	1:5,000	Gift from Miles Epstein	Fung et al., 2017
CT-B	Goat	1:1,000	List Biologicals; gift from Colin Anderson	Gwynne et al., 2009
**Secondary antibodies**		**Host**	**Dilution**	**Source**
Anti-goat AF594		Donkey	1:1,000	Molecular Probes
Anti-sheep AF647		Donkey	1:500	Molecular Probes
Anti-sheep AF488		Donkey	1:400	Molecular Probes
Anti-human AF647		Donkey	1:500	Jackson Immuno Labs
Anti-human AF488		Donkey	1:800	Jackson Immuno Labs

*ChAT, choline acetyltransferase; nNOS, neuronal nitric oxide synthase; VIP, vasoactive intestinal peptide; CT-B, cholera toxin B-subunit*.

### Confocal imaging and cell counts

Submucosal plexus and myenteric plexus preparations were imaged with a 40x objective and cryosections were imaged with a 20x objective using a Zeiss Pascal confocal microscope. The proportion of each neuronal subtype (ChAT^+^, nNOS^+^, calretinin^+^, pCREB^+^, and/or c-Fos^+^) was obtained by examining co-expression with at least 100 Hu^+^ submucosal neurons or 200 Hu^+^ myenteric neurons per preparation. The mean proportion of each neuronal subtype was determined by averaging the proportions obtained from three to six animals.

### Drugs

CT, 1,1-dimethyl-4-phenylpiperazinium (DMPP; Sigma Aldrich), hyoscine, and nicardipine were dissolved in distilled water to make stock solutions. Stocks were stored at 4°C, except nicardipine which was stored as aliquots at −20°C. Drugs were diluted in physiological saline to working concentrations when required.

### Statistics

Two-tailed unpaired *t*-tests were conducted to determine statistical significance, unless specified otherwise. *P* < 0.05 was considered significant. Analyses were performed using GraphPad Prism 5.0 (GraphPad softwares, San Diego, California, USA). Data are presented as mean ± the standard error of the mean (SEM), and “*n*” number of cells examined unless stated otherwise.

## Results

**Figure 1:**
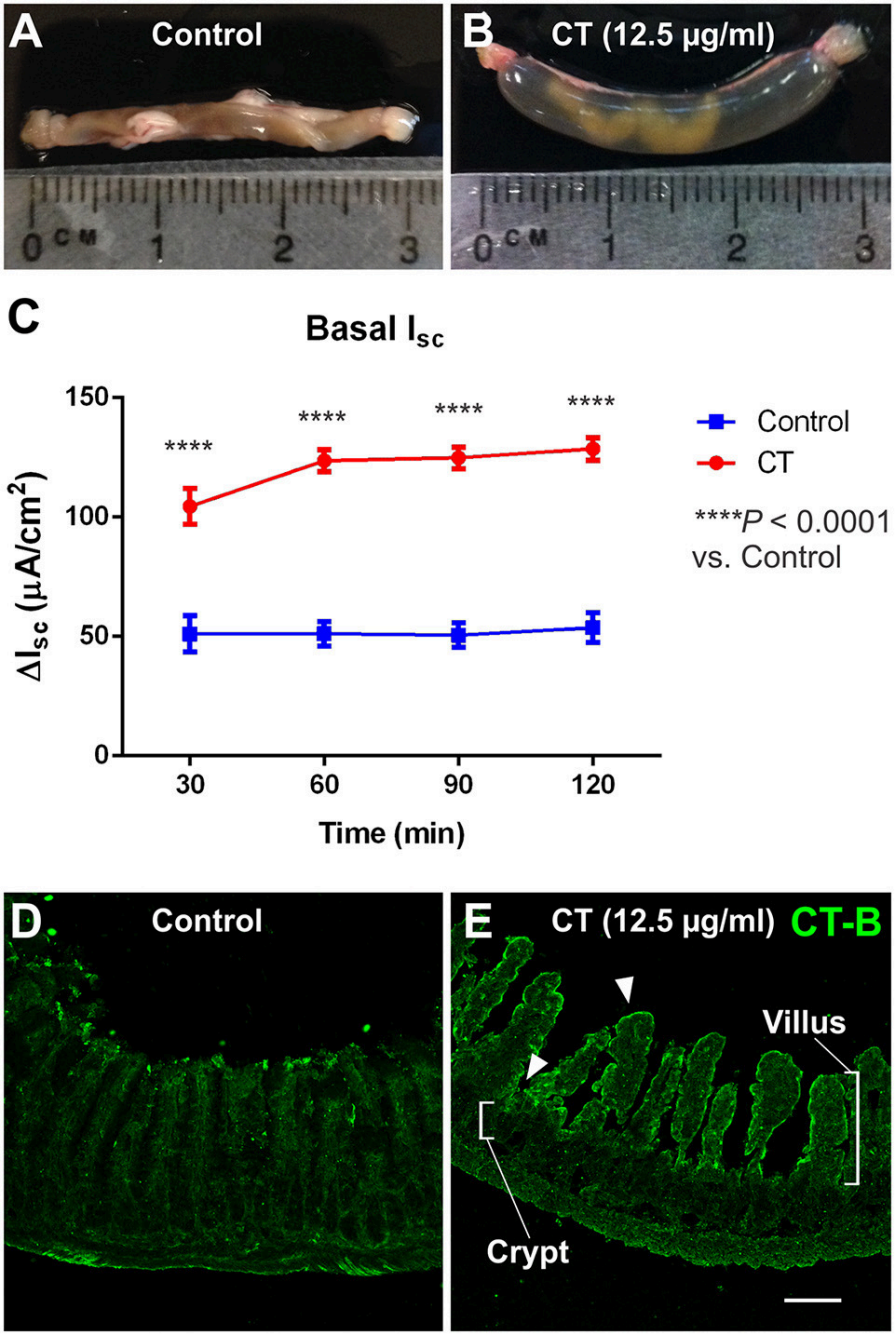
CT-treated vs. control ileal loops following 3.5 h incubation *in vivo*. Images of ileal loops after a 3.5 h incubation *in vivo* with saline (control, **A**), or CT (12.5 μg/ml, **B**) in saline. CT-treated loops displayed substantial accumulation of fluid that was absent in controls. Accordingly, basal I_SC_ measured in full thickness ileal tissues and monitored for 120 min following CT-incubation (*n* = 11) was significantly higher than that of saline controls (*n* = 10; two-way ANOVA with Bonferroni's post-test, ^****^P < 0.0001) **(C)**. Confocal micrographs of cryosections from control **(D)** and CT-treated ileal loops **(E)** fluorescently labeled for CT-B. CT-B staining was specifically observed in CT-treated tissues and was confined to the mucosal epithelium. CT-B-immunoreactivity was observed predominantly in the villi, but also close to the crypts, as indicated by arrowheads. Scale bar = 100 μm.

All ligated ileal loops that were incubated with CT (12.5 μg/ml; 3.5 h) displayed increased accumulation of fluid in the lumen compared to the saline controls (Figures 1A,B). We confirmed that this model of CT-incubation induced a sustained hypersecretion in full thickness preparations by monitoring basal short-circuit current (I_SC_) in Ussing chambers. The basal I_SC_ measured 30 min following mounting of CT-exposed tissues into the chambers was 105 ± 8 μA/cm^2^ (*n* = 11 mice), which was higher than in control tissues (51 ± 8 μA/cm^2^; *n* = 10 mice) (Figure 1C). Further, the basal I_SC_ of CT-treated tissues remained significantly higher than control levels over 120 min of recording (two-way ANOVA with Bonferroni's post-test, *P* < 0.0001). As previously reported for the guinea pig jejunum, anti-CT-B labeling was only observed in CT-treated tissues and was confined to the mucosal epithelium (Figures 1D,E) (Gwynne et al., 2009). Notably, CT-B-immunoreactivity was observed predominantly in the villi, but also close to the crypts (Figure 1E).

### CT-incubation induced pCREB expression in the submucosal plexus

**Figure 2:**
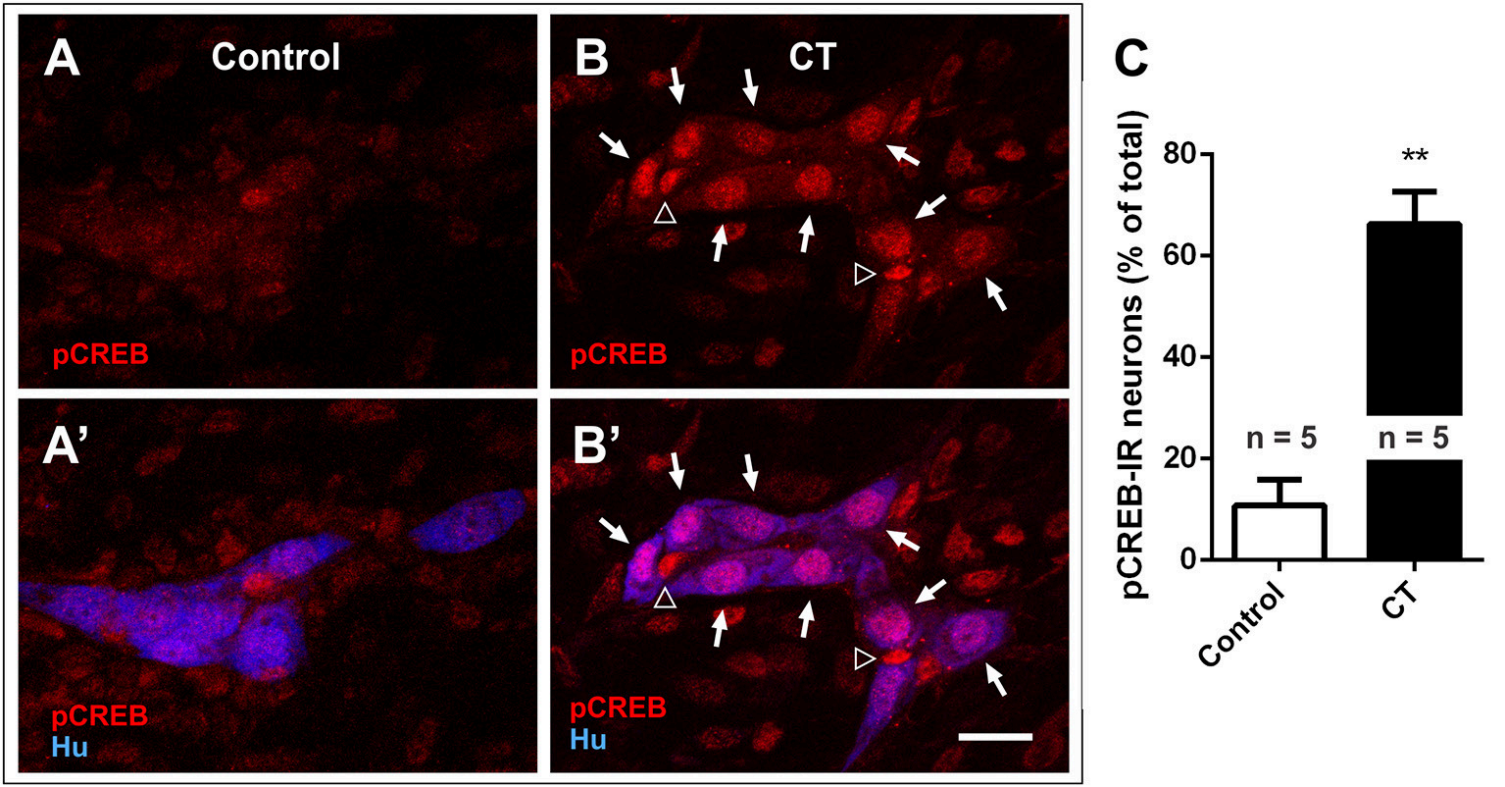
Nuclear pCREB expression increased in the submucosal plexus following CT-incubation. Confocal images of submucosal ganglia from a control **(A,A′)** and a CT-treated ileal loop **(B,B′)**, labeled for pCREB (red) and the neuronal marker Hu (blue). Scale bar = 20 μm. Neuronal (Hu^+^, arrows) and possibly glial (Hu^−^, empty arrowheads) pCREB^+^ nuclei were observed within the ganglia of CT-incubated preparations. **(C)** A significantly higher proportion of submucosal neurons (Hu^+^) displayed nuclear pCREB staining after CT-treatment compared to controls (^**^P < 0.01).

We first examined the nuclear expression of two activity-dependent markers, pCREB and c-Fos, in the submucosal plexus of CT-incubated and control preparations to reveal submucosal neurons that are activated during CT incubation (Figures 2A–C) (Sheng and Greenberg, 1990; Kirchgessner et al., 1992). Higher levels of pCREB expression were observed in CT-incubated preparations, where 65 ± 7% of all submucosal neurons were pCREB^+^ (*n* = 3 mice, 413 Hu^+^ neurons examined), while only 11 ± 5% were pCREB^+^ in controls (*n* = 3 mice; 390 Hu^+^ neurons examined; *P* < 0.01) (Figure 2C). A significant number of pCREB^+^/Hu^−^ nuclei were also observed within submucosal ganglia, suggesting that CT-incubation possibly activated glial cells (Figures 2B,B′).

No c-Fos expression was observed in neurons of the submucosal plexus (**Figure 5E,E′**), although the conditions under which c-Fos expression is induced in submucosal neurons of mouse are unclear as this has not been previously examined.

### CT-exposure induced sustained excitability in cholinergic submucosal neurons

**Figure 3:**
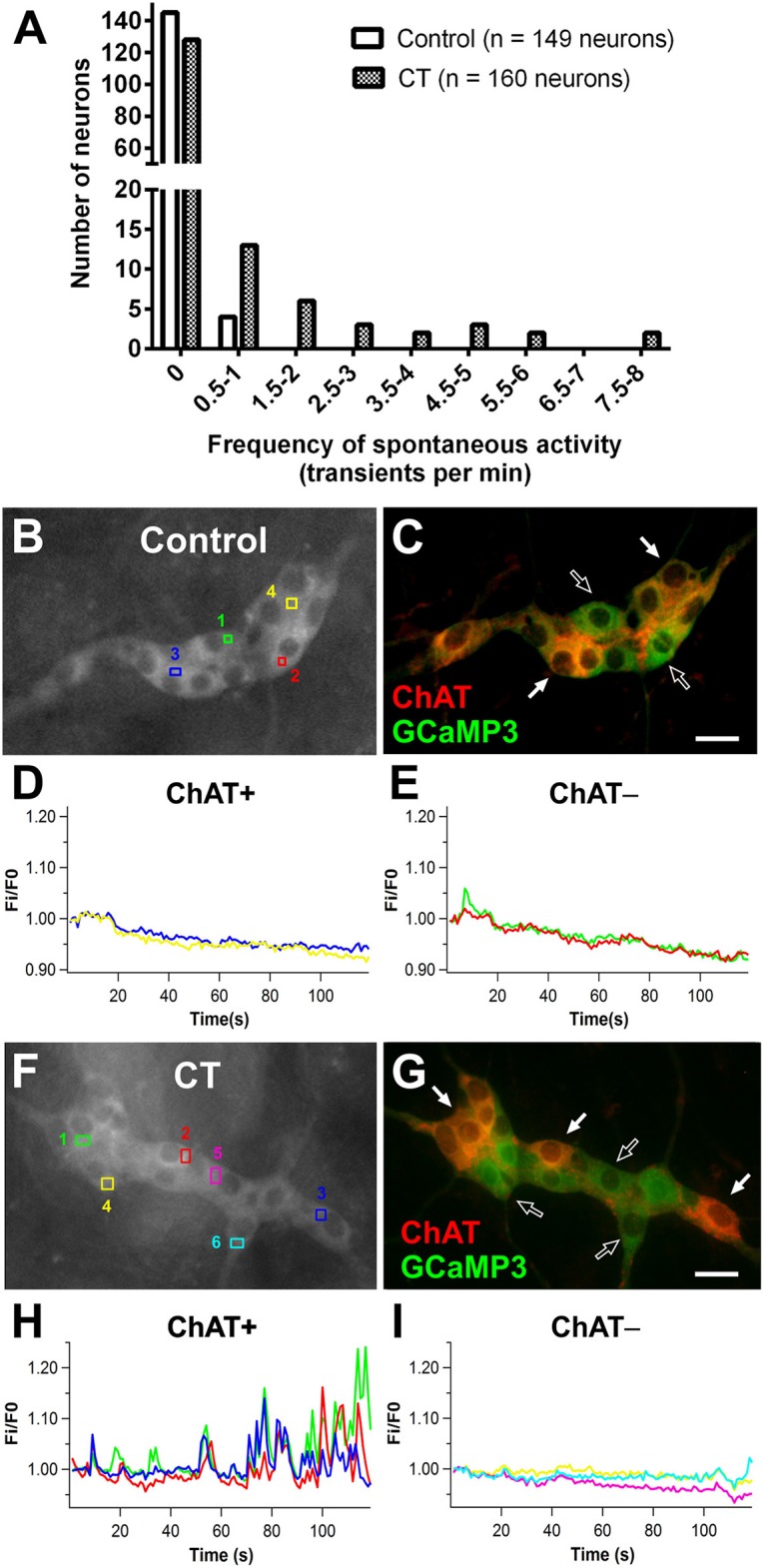
Spontaneous Ca^2+^ activity in submucosal neurons increased following CT-incubation. **(A)** The frequency of spontaneous [Ca^2+^]_i_ transients in submucosal neurons of control vs. CT-treated tissues (*n* = 12 ganglia examined per condition). Spontaneous activity was rarely observed in control preparations **(B–E)**, whereas submucosal neurons of CT-treated preparations displayed significant levels of spontaneous activity. **(F)** A submucosal ganglion that showed spontaneous activity following CT-exposure. **(G)** These neurons were identified as cholinergic neurons using *post-hoc* staining for choline acetyltransferase (ChAT, red). **(H)** Traces from spontaneously active ChAT^+^ neurons as marked by filled arrows in **(G)**. Traces are color-coded to correspond with selected regions of interest in **(F)**. No spontaneous [Ca^2+^]_i_ transients were observed in ChAT^−^ neurons within the same ganglion **(I)** as indicated by unfilled arrows in **(G)**. Scale bars = 20 μm.

Using Ca^2+^ imaging, submucosal plexus preparations from ileal loops of Wnt1-*Cre;R26R*-GCaMP3 mice were first imaged for 2 min without external stimulation to examine spontaneous activity. Submucosal neurons rarely displayed spontaneous [Ca^2+^]_i_ transients in control preparations (4/149 neurons were spontaneously active) (Figures 3A–E). However, a significantly larger proportion of submucosal neurons (32/160 neurons; Figure 3A) displayed spontaneous [Ca^2+^]_i_ transients in CT-incubated preparations (Fisher's exact test; *P* < 0.0001). Spontaneously active neurons identified by *post-hoc* immunostaining were exclusively cholinergic (ChAT^+^; Figures 3F–I).

**Figure 4:**
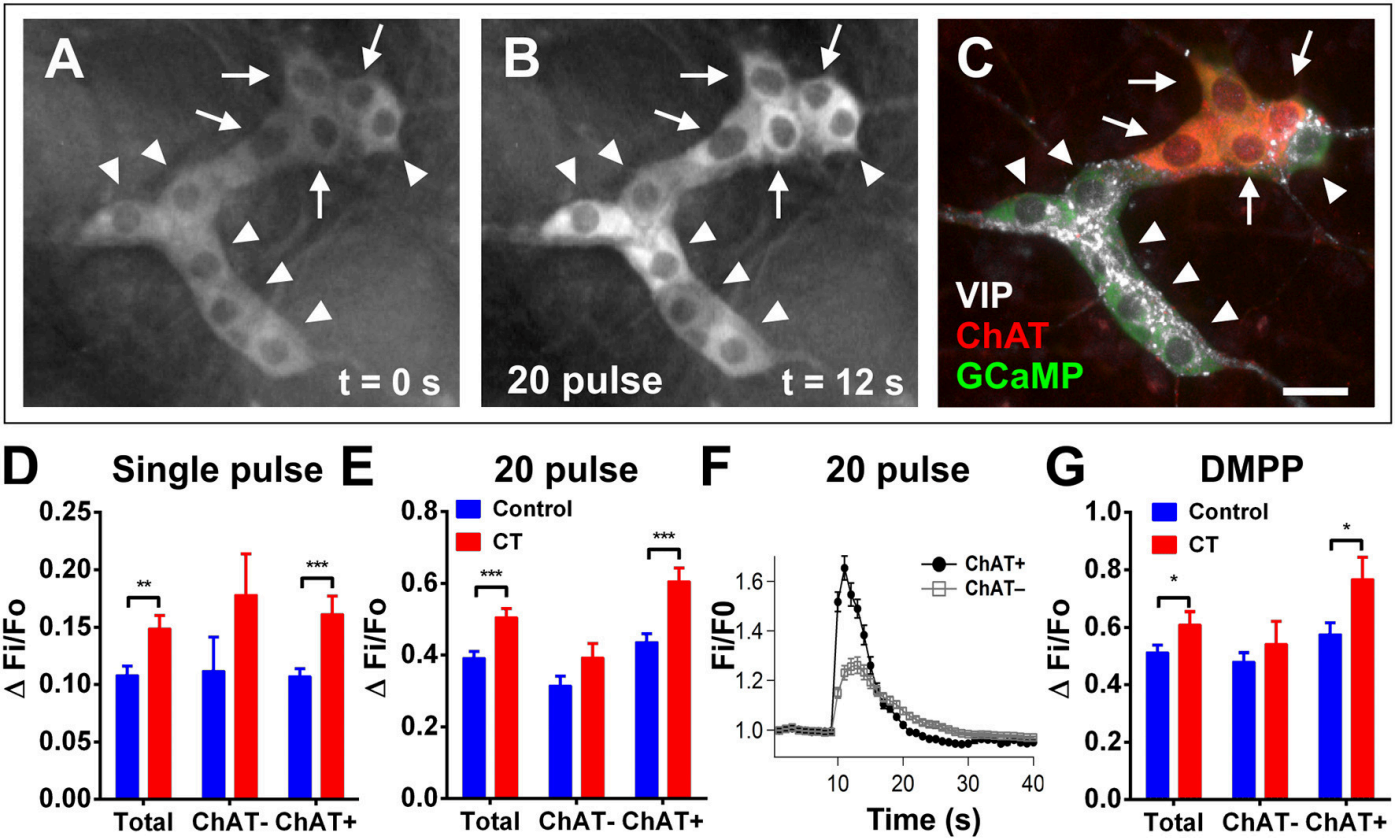
Electrically- and DMPP-evoked [Ca^2+^]_i_ responses in submucosal neurons are increased following CT-exposure. **(A–C)** A submucosal ganglion from a CT-incubated preparation responding to a train of stimuli (20 pulse). Cholinergic neurons were identified with *post-hoc* labeling for choline acetyltransferase (ChAT, red); non-cholinergic neurons were vasoactive intestinal peptide (VIP)-immunoreactive (white) **(C)**. Arrows indicate cholinergic ChAT^+^ neurons and arrowheads indicate VIP^+^ neurons. Scale bar = 20 μm. In each histogram **(D,E,G)** the amplitude (ΔFi/F0) of “Total” neurons that responded was presented (left) then divided into those of ChAT^−^ (middle) and ChAT^+^ (right) groups. Electrically-evoked responses (**D** single pulse and **E** 20 pulse) of submucosal neurons after CT-incubation had a higher amplitude compared to that of controls (single pulse: ^**^P < 0.01, control: *n* = 43 neurons, CT: *n* = 39 neurons; 20 pulse: ^***^P < 0.001, control: *n* = 92 neurons, CT: *n* = 64 neurons), and this was specifically observed in cholinergic neurons (^***^P < 0.001). **(F)** Averaged traces of responses to 20 pulse stimulation (applied at 10 s) in ChAT^+^ (black trace) vs. ChAT^−^ neurons (gray trace; mean ± SEM) in a CT-treated preparation, as depicted in **(A–C)**. **(G)** Overall responses to DMPP were significantly higher after CT-treatment (^*^P < 0.05; control: *n* = 91 neurons, CT: *n* = 60 neurons), and in particular, responses in cholinergic (ChAT^+^) submucosal neurons were significantly increased (^*^P < 0.05; control: *n* = 36 neurons, CT: *n* = 22 neurons).

To then assess any changes to the excitability of the neural network, submucosal ganglia were stimulated electrically and chemically (Figure 4). Electrical stimulation (single pulses and trains of stimuli) evoked significantly larger [Ca^2+^]_i_ responses in CT-incubated submucosal plexus preparations compared to controls. A single pulse stimulated a larger response amplitude in submucosal neurons of CT-treated preparations (CT: ΔFi/F0 = 0.15 ± 0.01, *n* = 39; controls: ΔFi/F0 = 0.11 ± 0.01, *n* = 46; *P* = 0.004; Figure 4D). Using *post-hoc* labeling of imaged ganglia, ChAT^+^ neurons specifically displayed larger responses (CT: ΔFi/F0 = 0.16 ± 0.02, *n* = 22; control: ΔFi/F0 = 0.11 ± 0.01, *n* = 36; *P* = 0.0006). Whereas no significant differences were observed in ChAT^−^ neurons (*P* = 0.23; CT: *n* = 4; control: *n* = 10). Similarly, trains of stimuli evoked larger responses after CT-incubation (CT: ΔFi/F0 = 0.51 ± 0.02, *n* = 64 vs. control: ΔFi/F0 = 0.39 ± 0.02, *n* = 92; *P* = 0.0003; Figure 4E) and this was also specific to cholinergic neurons (CT: ΔFi/F0 = 0.61 ± 0.04, *n* = 24; control: ΔFi/F0 = 0.44 ± 0.02, *n* = 58; *P* = 0.0002; Figures 4A–C,E,F). No differences were observed ChAT^−^ neurons (*P* = 0.093; CT: *n* = 20; control: *n* = 34). We stained some submucosal preparations for VIP and ChAT and found that responding neurons were either VIP^+^ or ChAT^+^ but not both or neither (Figure 4C). Hence, ChAT^−^ neurons that responded to CT are assumed to be VIP^+^ neurons.

Application of the nicotinic agonist 1,1-dimethyl-4-phenylpiperazinium (DMPP; 10 μM; nicotinic agonist) by pressure ejection from a pipette located close to a selected ganglion also evoked [Ca^2+^]_i_ transients with significantly higher response amplitudes (ΔFi/F0) in CT-exposed compared to control tissues (CT: ΔFi/F0 = 0.61 ± 0.05, *n* = 60; control: ΔFi/F0 = 0.51 ± 0.02, *n* = 91; *P* = 0.044; Figure 4G). Again, ChAT^+^ neurons specifically displayed significantly larger responses to DMPP in CT-exposed tissues compared to controls (*P* = 0.017; CT: *n* = 22; control n: = 42). Ca^2+^ transients seen in ChAT^−^ neurons did not differ significantly between CT and saline-incubated preparations (*P* = 0.40; CT: *n* = 22; control: *n* = 39).

### CT-exposure induces c-Fos expression in myenteric plexus

**Figure 5:**
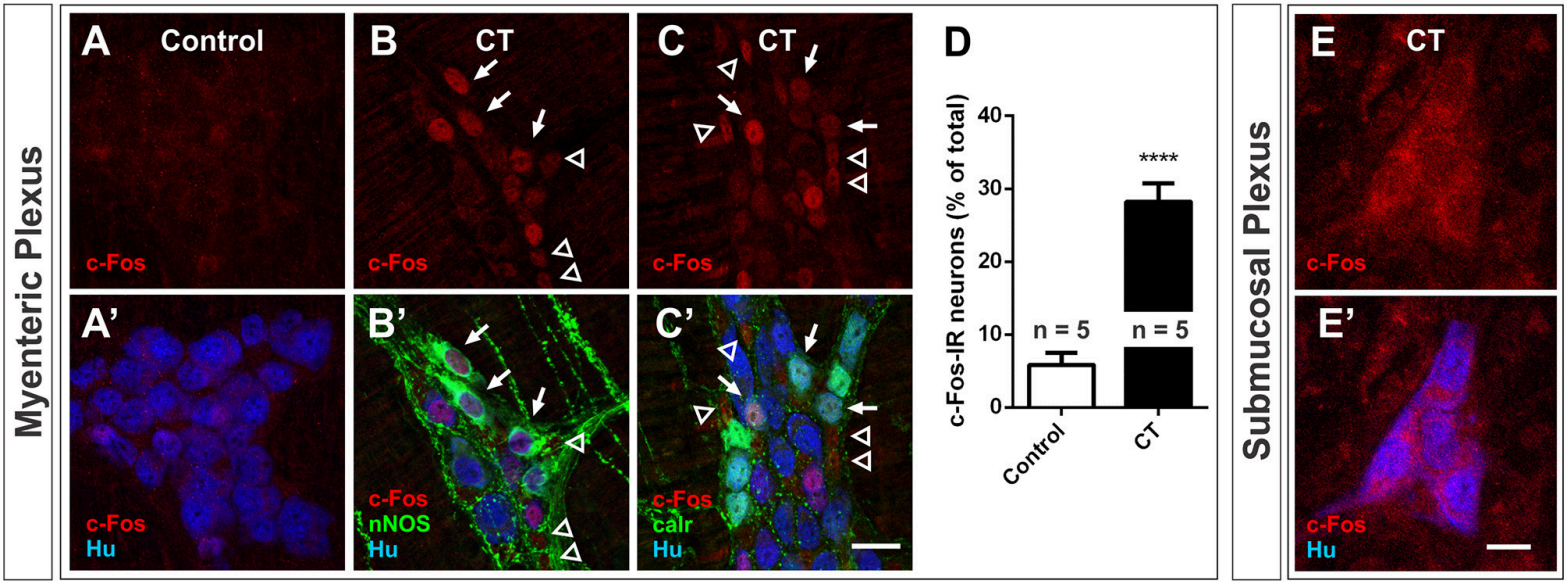
Nuclear c-Fos expression in the myenteric plexus increased following CT-incubation. Confocal images of myenteric ganglia from a control **(A,A′)** and a CT-treated ileal loop **(B–C**′**)**, labeled for c-Fos (red) and the neuronal marker Hu (blue). Some CT-treated preparations were co-labeled with neuronal nitric oxide synthase (nNOS, **B**′) and some were co-labeled with calretinin (calr, **C**′). Arrows indicate Hu^+^ neurons displaying nuclear c-Fos labeling and nNOS or calretinin expression. Empty arrowheads mark possible glial (Hu^−^) nuclei displaying c-Fos expression. **(D)** A significantly higher proportion of myenteric neurons (Hu^+^) displayed nuclear c-Fos labeling after CT-treatment compared to controls (^****^P < 0.0001; *n* = no. of animals examined). **(E,E′)** We did not observe any nuclear cfos labeling in submucosal ganglia. Scale bars = 20 μm.

The expression of pCREB and c-Fos was also examined in the myenteric plexus of CT-incubated and control preparations. We observed a significant increase in c-Fos expression in the myenteric plexus in CT incubated preparations compared to controls: 28 ± 3% of myenteric neurons displayed c-Fos-immunoreactivity (*n* = 5 mice; 2644 Hu^+^ neurons examined), compared to 6 ± 2% of myenteric neurons in controls (*n* = 5 mice; 2438 Hu^+^ neurons examined; *P* < 0.0001) (Figures 5A–D). Given that c-Fos expression was observed in a sub-population of myenteric neurons, antisera against the neurochemical markers, nNOS and calretinin were used to determine if specific functional subtypes were activated during the CT-treatment (Figures 5B,C). Together, these two distinct neurochemical classes (nNOS and calretinin) represent a large majority of myenteric neurons (Furness, 2000; Qu et al., 2008). nNOS^+^ neurons include inhibitory motor neurons and/or interneurons, while calretinin^+^ neurons include intrinsic sensory neurons, excitatory motor neurons and/or interneurons (Qu et al., 2008). c-Fos expression was mainly observed in nNOS^+^ neurons (63 ± 5% of c-Fos^+^ neurons were nNOS^+^ and 56 ± 6% of nNOS^+^ neurons were c-Fos^+^; *n* = 4 mice; Figures 5B,B′). The remaining c-Fos^+^ neurons were mostly calretinin^+^: 30 ± 1% of c-Fos^+^ neurons were calretinin^+^ (19 ± 2% of calretinin^+^ neurons were c-Fos^+^; *n* = 3 mice; Figures 5C,C′). c-Fos-immunoreactivity was also observed in many Hu^−^ cells within the myenteric ganglia (Figures 5B,C). pCREB expression in the myenteric plexus was profuse even in control preparations and hence was not a useful marker.

### The myenteric plexus displays an overall reduction in neuronal excitability following CT-exposure

In contrast to the submucosal plexus, a smaller proportion of myenteric neurons displayed spontaneous [Ca^2+^]_i_ transients in CT-treated preparations (1/263 neurons were spontaneously active) compared to controls (14/293 neurons; Fisher's exact test *P* < 0.01).

**Figure 6:**
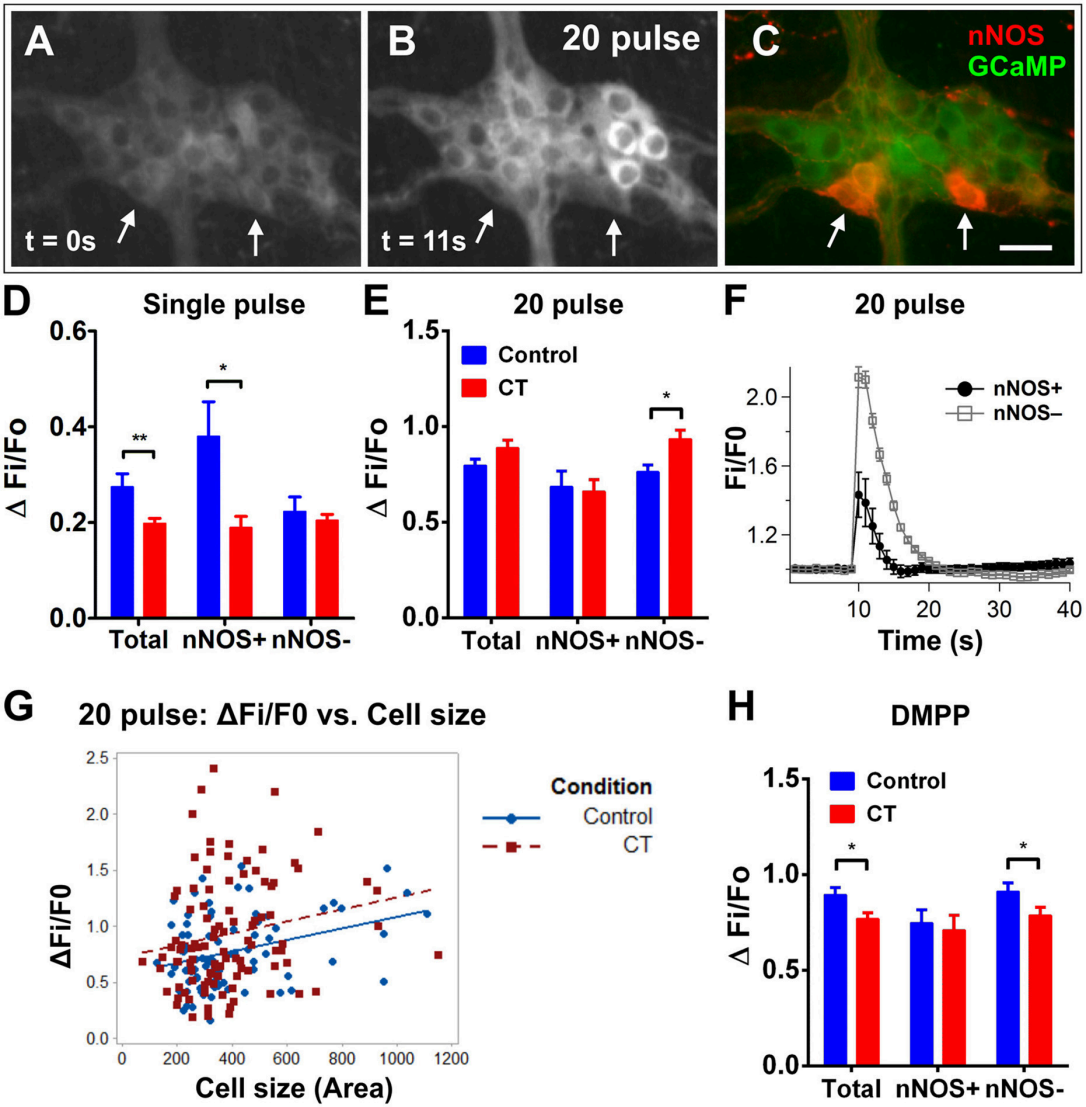
Electrically- and DMPP-evoked [Ca^2+^]_i_ responses in myenteric neurons are decreased following CT-exposure. **(A–C)** A myenteric ganglion from a CT-incubated preparation responding to a train of stimuli (20 pulse). Cholinergic neurons were identified with *post-hoc* labeling for nNOS (neuronal nitric oxide synthase; red, **C**). Arrows indicate the nNOS^+^ neurons that responded. Scale bar = 20 μm. In each histogram **(D,E,H)** the amplitude (ΔFi/F0) of “Total” neurons that responded was presented (left) then divided into those of nNOS^−^ (middle) and nNOS^+^ (right) groups. **(D)** Single pulse stimuli also evoked smaller amplitude responses after CT-incubation (^**^P < 0.01; control: *n* = 62 neurons, CT: *n* = 84 neurons) and this was specifically observed in nNOS^+^ neurons (^*^P < 0.05). **(E)** Overall responses to 20 pulse stimulation were not different after CT-exposure (control: *n* = 131 neurons, CT: *n* = 112 neurons). However, a slight but significant increase (*P* < 0.05) was observed in nNOS^−^ neurons. **(F)** Averaged traces of responses to 20 pulse stimulation (applied at 10 s) in nNOS^+^ (black trace) vs. nNOS^−^ neurons (gray trace; mean ± SEM) from a CT-treated preparation, as depicted in **(A–C)**. **(G)** The size (area) of nNOS^−^ neuronal cell bodies were plotted against corresponding 20 pulse stimulated response amplitudes to examine if select populations of nNOS^−^ neurons may be differentially affected by CT treatment. Multiple linear regression analysis showed no significant differences in the correlation between cell size and response amplitudes in control vs. CT conditions (control: *n* = 80 nNOS^−^ neurons examined; CT: *n* = 101 nNOS^−^ neurons examined). **(H)** Overall DMPP (10 μM) response amplitudes were significantly smaller after CT-treatment (control: *n* = 146 neurons, CT: *n* = 152 neurons), in particular nNOS^−^ myenteric neurons were significantly decreased (^*^P < 0.05).

Stimulating electrically with a single pulse evoked smaller responses in myenteric neurons of CT-treated preparations (CT: ΔFi/F0 = 0.19 ± 0.01, *n* = 84; control: ΔFi/F0 = 0.28 ± 0.03, *n* = 62; *P* = 0.002; Figure 6D). This effect was observed in nNOS^+^ neurons (CT: ΔFi/F0 = 0.19 ± 0.02, *n* = 8; control: ΔFi/F0 = 0.41 ± 0.07, *n* = 9; *P* = 0.016), rather than nNOS^−^ neurons. In contrast, nNOS^−^ neurons appear to show a significantly increased response to the train of stimuli after CT-exposure (CT: ΔFi/F0 = 0.92 ± 0.05, *n* = 101; control: ΔFi/F0 = 0.76 ± 0.04, *n* = 81; *P* = 0.012; Figures 6A–C,E,F), while responses of nNOS^+^ neurons did not differ. This difference was not apparent when comparing the overall response amplitude of all neurons responding (CT: ΔFi/F0 = 0.87 ± 0.04, *n* = 131; control: ΔFi/F0 = 0.80 ± 0.03, *n* = 112; *P* = 0.17).

Given that nNOS^−^ neurons specifically displayed increased responses to train simulation following CT-incubation, we further investigated whether select subpopulations of nNOS^−^ neurons may have been differentially affected by CT-exposure. Some neuronal subtypes may be deduced by their cell size. For instance, the cell bodies of intrinsic sensory neurons are nNOS^−^ and are typically larger in size compared with other subtypes (Qu et al., 2008). Thus, we measured the size (area) of nNOS^−^ cells that responded to 20 pulse stimulation and plotted this against their corresponding response amplitude (Figure 6G). Using multiple linear regression analysis, we compared the regression coefficients between CT and control conditions, but no significant differences were found.

Further, a reduction in the amplitude of [Ca^2+^]_i_ responses to DMPP **(**10 μM) was observed in CT-treated preparations compared to controls (CT: ΔFi/F0 = 0.77 ± 0.03, *n* = 152; control: ΔFi/F0 = 0.88 ± 0.04, *n* = 146; *P* = 0.028; Figure 6H). As a large proportion of c-Fos^+^ neurons were nNOS^+^, the responding neurons were divided into nNOS^+^ and nNOS^−^ groups using *post-hoc* immunostaining. nNOS^−^ neurons were found to display smaller responses after CT-treatment (CT: ΔFi/F0 = 0.79 ± 0.04, *n* = 105; control: ΔFi/F0 = 0.91 ± 0.04, *n* = 119, *P* = 0.044), while responses in nNOS^+^ neurons were unchanged (CT: ΔFi/F0 = 0.71 ± 0.08, *n* = 29; control: ΔFi/F0 = 0.75 ± 0.07, *n* = 27, *P* = 0.73).

**Figure 7:**
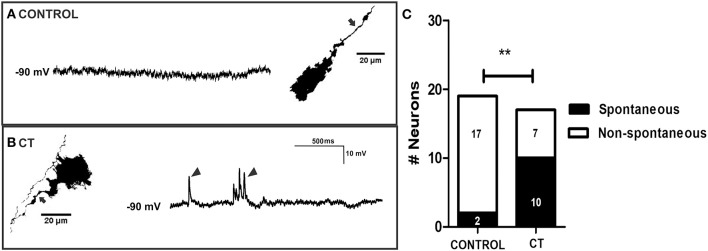
Myenteric neurons of CT-incubated ileal segments displayed increased spontaneous firing. Representative micrographs showing myenteric neurons that were injected with biocytin (axons indicated by arrows) during recording and their corresponding intracellular recordings from control **(A)** and CT-treated **(B)** preparations. Myenteric neurons showed an increase in spontaneous activity (single and bursts of fast EPSPs indicated by arrow heads) following CT exposure. **(C)** Graphical representation of the significantly higher proportion of myenteric neurons that displayed spontaneous firing of fast EPSPs after CT-treatment. Numbers of spontaneously active neurons are displayed within each histogram (^**^P < 0.01).

The overall reduction in calcium activity in the myenteric neurons observed in the present study contrasts with our recent report on the guinea-pig jejunum, where a subset of myenteric neurons exhibited sustained hyperexcitability after exposure to CT *in vitro* (Koussoulas et al., 2017). Accordingly, the activity of a group of myenteric neurons was recorded via intracellular recording. CT treatment did not have any obvious effects on the activity of myenteric neurons when recorded at their resting membrane potentials (control *n* = 8; CT *n* = 6). At a hyperpolarized membrane potential (-90 mV), which increased the sizes of the fast EPSPs, the amplitudes of electrically-evoked fast EPSPs did not differ between CT (28.6 ± 2.6 mV, *n* = 12) and controls (23.6 ± 2.9 mV; *n* = 13, *P* = 0.22). However, a greater proportion of myenteric neurons displayed an increase in basal neuronal excitability (single and bursts of spontaneous fast EPSPs) following CT exposure (CT: 10 of 17 neurons; control: 2 of 19 neurons; Fisher's exact test, *P* < 0.01; Figures 7A–C). Thus, heightened synaptic activity within the myenteric circuitry was revealed at hyperpolarized potentials. However, taken together with our Ca^2+^ imaging results, the role of myenteric neurons remains inconclusive.

### Effects of CT were confined to the ileal loop

**Figure 8:**
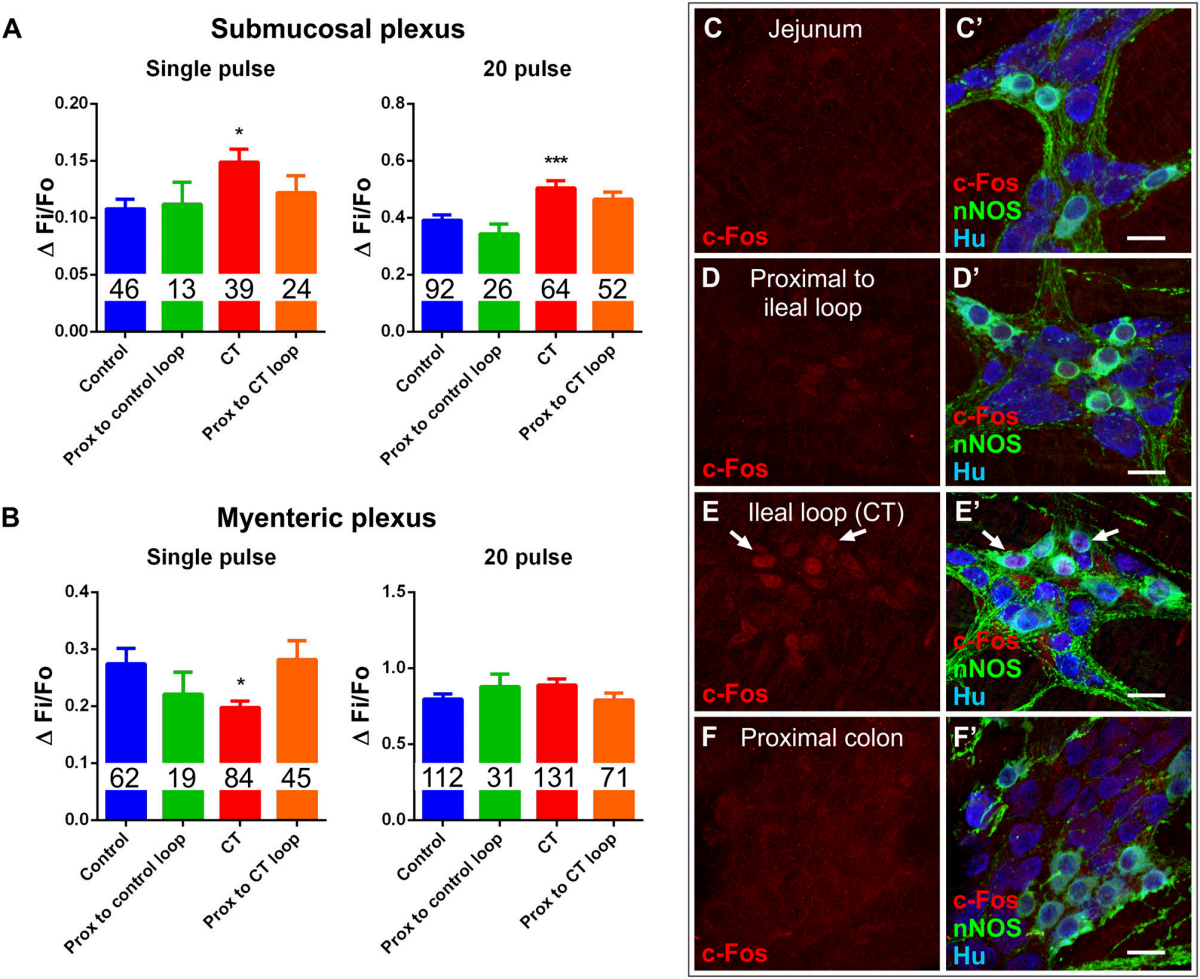
Effects of CT-exposure were confined to the ileal loop. **(A)** [Ca^2+^]_i_ responses of submucosal neurons to electrical stimulation (single pulse and 20 pulse) in segments of ileum proximal to the control loop or proximal to the CT-treated loop did not differ from controls (^*^P < 0.05, ^***^P < 0.0001 vs. control). **(B)** Response amplitudes (ΔFi/F0) of myenteric neurons to electrical stimulation (single pulse and 20 pulse) in segments of ileum proximal to the control loop or proximal to the CT-treated loop also did not differ from controls (^*^P < 0.05 vs. control). Number of neurons examined are displayed within the bars. Confocal images of myenteric ganglia from jejunum **(C,C′)**, a segment proximal to the ileal loop **(D,D′)**, proximal colon **(F,F′)**, and the CT-treated ileal loop **(E,E′)**, labeled for c-Fos (red), nNOS (green), and the neuronal marker Hu (blue). Arrows indicate neurons (Hu^+^) displaying colocalization of c-Fos- and nNOS-immunoreactivity. Nuclear c-Fos labeling was confined to the CT-treated ileal loop and was not observed in other intestinal regions. Scale bars = 20 μm.

Some bacterial toxins have off-target effects, for instance, *Clostridium difficile* toxin A activates nerve reflexes involving extrinsic neurons to induce widespread inflammatory and secretory responses (Pothoulakis and Lamont, 2001; Spiller, 2002). The potential for off-target effects of CT was assessed in intestinal segments outside the ileal loop. Ileal segments proximal to the CT-treated loop were examined in Ussing chambers. Basal I_SC_ in these tissues was 43 ± 9 μA/cm^2^ (*n* = 9 mice) and was not different from that of control ileal loops (51 ± 8 μA/cm^2^; *n* = 10 mice) or ileal segments proximal to the control ileal loop (33 ± 18 μA/cm^2^; *n* = 4 mice). Furthermore, electrical stimulation (single pulse, and a train of stimuli) of submucosal plexus preparations evoked neuronal [Ca^2+^]_i_ responses in ileal segments proximal to the CT-treated loop (single pulse: ΔFi/F0 = 0.12 ± 0.01, *n* = 24; 20 pulses: ΔFi/F0 = 0.46 ± 0.03, *n* = 52) and proximal to control loops (single pulse: ΔFi/F0 = 0.11 ± 0.02, *n* = 13; 20 pulses: ΔFi/F0 = 0.34 ± 0.04, *n* = 26) that did not differ from those of control loops (Figure 8A). Similarly, electrical stimulation of myenteric plexus preparations also evoked neuronal [Ca^2+^]_i_ responses in ileal segments proximal to the CT-treated loop (single pulse: ΔFi/F0 = 0.28 ± 0.03, *n* = 45; 20 pulses: ΔFi/F0 = 0.79 ± 0.05, *n* = 71) and proximal to control loops (single pulse: ΔFi/F0 = 0.22 ± 0.04, *n* = 19; 20 pulses: ΔFi/F0 = 0.88 ± 0.08, *n* = 31) that did not differ from those of control loops (Figure 8B). Additionally, c-Fos labeling was observed to be confined to the CT-incubated ileal loop, with no labeling in the jejunum, the region just proximal to the ileal loop, or the region distal to the ileal loop (proximal colon) of these mice (*n* = 4) (Figures 8C–F′), suggesting that CT does not have any off-target effects in this preparation.

## Discussion

### CT induced sustained hyperexcitability in cholinergic submucosal neurons

We found increased neuronal excitability and spontaneous activity in the submucosal plexus following CT-incubation. Following CT incubation, it was specifically cholinergic submucosal neurons that displayed an increase in spontaneous [Ca^2+^]_i_ transients and in [Ca^2+^]_i_ responses to electrical and chemical stimuli. While CT-B may directly bind submucosal VIP neurons in guinea pig (Jiang et al., 1993), we found that CT-B labeling was restricted to the mucosal epithelium and did not reach the underlying enteric plexuses. Hence, it is unlikely that CT directly or specifically binds cholinergic neurons in our model.

Much of the previous literature highlights a role for VIP, a potent secretagogue, and VIP-containing secretomotor neurons in various species including rats, guinea pigs, cats, and humans (Bloom et al., 1976; Cassuto et al., 1981; Mourad et al., 1995; Banks et al., 2005; Kordasti et al., 2006; Gwynne et al., 2009). Although there is considerable evidence in rats showing that VIP antagonists can attenuate CT-evoked hypersecretion, the involvement of VIP has not been completely clear due to discrepancies between studies in the efficacy of different VIP antagonists despite using the same animal model (Mourad and Nassar, 2000; Banks et al., 2005; Kordasti et al., 2006). Our finding that CT induces sustained changes in cholinergic submucosal neurons may reflect differences in the enteric circuitry of mice compared to that of larger mammals. Nevertheless, we may in fact reveal a novel sustained increase in Ca^2+^ activity in cholinergic neurons that can contribute to CT effects or even render the ENS more susceptible to future insults.

VIP is associated with a cAMP-mediated sustained Cl^−^ secretory response (Burleigh and Banks, 2007; Xue et al., 2007), while ACh evokes a rapid, but transient, Ca^2+^-mediated Cl^−^ secretory response (Hirota and McKay, 2006). The effects of ACh are limited by intracellular signaling mechanisms within epithelial cells (Keely and Barrett, 2000) that uncouple [Ca^2+^]_i_ increases and Cl^−^ secretion; this inhibition can last for over 90 min (Vajanaphanich et al., 1994). Although CT pre-exposure can potentiate ACh-evoked secretion, even potentiated responses of ACh remain short-lived (Banks et al., 2004). It is therefore surprising that CT induced a sustained increase in the excitability of cholinergic secretomotor neurons. On the other hand, perhaps it is more appropriate for the ENS to limit the secretory response to minimize further loss of fluid and electrolytes as it attempts to flush out the toxin.

The role of cholinergic pathways within the circuitry in CT-induced effects remains unclear. Nicotinic transmission may be involved in maintaining the established CT-hypersecretory response, but not in its induction (Kordasti et al., 2006). Blocking nicotinic receptors, but not muscarinic receptors, inhibits CT-evoked hypersecretion (Cassuto et al., 1982; Kobayashi et al., 2001; Kordasti et al., 2006). Our computational modeling data of the guinea-pig submucous plexus circuitry suggests that this could be due to cholinergic neurons forming recurrent networks to reinforce activity within the circuitry via nAChR-mediated fast EPSPs (Chambers et al., 2005). Hence, CT may increase the excitability of these interconnected cholinergic submucosal neurons (Reed and Vanner, 2001) through nicotinic transmission to increase the output secretory response. However, without slow excitatory inputs from sensory and/or VIP neuron networks that are necessary for sustaining hyperactivity in the circuitry, nAChR activation only evokes transient effects (Chambers et al., 2005).

The fluid secreted across the epithelium is partly drawn from the circulatory system; accordingly, the control of blood flow and secretion are closely linked (Furness, 2006). As intrinsic submucosal vasodilation is predominantly cholinergic (Neild et al., 1990; Vanner et al., 1993; Vanner and Surprenant, 1996), an increase in the excitability of cholinergic vasodilator neurons can contribute to CT-evoked hypersecretion *in vivo* by increasing local blood flow. Indeed, CT-incubation leads to a substantial increase in submucosal blood flow in cat small intestine (Cedgård et al., 1978).

### Increased pCREB stain reveals that most submucosal neurons are activated during CT-incubation

Our Ca^2+^ imaging data only revealed changes in the excitability of cholinergic submucosal neurons following CT-exposure. However, increased pCREB expression in the submucosal plexus indicates an increase in neuronal activity in both cholinergic and non-cholinergic neurons during CT-incubation. This suggests that both VIP and cholinergic submucosal neurons were active during CT-incubation, but only cholinergic submucosal neurons display increased excitability following CT exposure. Hence, VIP secretomotor neurons may be involved in the induction of the hypersecretion, rather than maintaining hypersecretion. In support of this, it has been reported that VIP antagonism effectively prevents CT-induced hypersecretion, but is ineffective once hypersecretion had established (Kordasti et al., 2006). It is also possible that changes in the excitability of VIP secretomotor neurons are not reflected in terms of Ca^2+^ signals, but depend on other secondary messenger systems, namely cAMP. CT is well-known to stimulate adenylyl cyclase and cAMP production at the level of the mucosal epithelium and evoke the release of intermediate signaling mediators, such as 5-HT, that can in turn activate enteric neurons (Beubler et al., 1989; Mourad et al., 1995). Indeed, the increase in pCREB labeling indicates that cAMP signaling is altered in submucosal neurons by CT exposure.

Co-incubation of CT with a VIP antagonist would be ideal to examine the involvement of VIP. Unfortunately, this approach is constrained by the VIP antagonists currently available, which are peptides with a relatively short half-life (Couvineau and Laburthe, 2012). In our closed ileal loop system, it is likely that the VIP antagonist will be degraded during the 3.5 h incubation.

There may also be increased glial activity within submucosal ganglia during CT incubation as we observed intraganglionic Hu- nuclei that were labeled with pCREB, which are likely glia (Gulbransen and Sharkey, 2012; Boesmans et al., 2015b). Interestingly, recent findings suggest that selective glial activation can stimulate electrogenic secretion through neurons in mouse colon (Grubišić and Gulbransen, 2017).

The other neuron activity marker, c-Fos expression was not observed in submucosal neurons in either the control or CT condition. pCREB is mainly associated with G-protein coupled receptor activation and increased cAMP, while c-Fos expression is associated with Ca^2+^-influx through voltage-gated Ca^2+^ channels; both are well-established activity-dependent markers (Kirchgessner et al., 1992; Ritter et al., 1997; Gammie and Nelson, 2001; Chen et al., 2007). The differences between c-Fos and pCREB labeling were surprising as pCREB can act upstream of c-Fos (Sheng and Greenberg, 1990; Sheng et al., 1991; Ginty et al., 1992). However, it may be that c-Fos expression is transient or that the intensity of the stimulus is insufficient to evoke c-Fos expression in submucosal neurons, whereas pCREB expression is observed due to its lower activation threshold (Fields et al., 1997). By contrast, CT induces c-Fos expression in both plexuses in guinea pig (Kirchgessner et al., 1992); this discrepancy may be attributed to species differences.

### Some myenteric neurons may be hyperactive during, but not following, CT incubation

CT-exposure did not evoke obvious changes in the excitability of myenteric neurons. However, myenteric c-Fos expression was increased in a select population of neurons (~30%), which includes nNOS^+^- and calretinin^+^-motor neurons and interneurons. An overall depression of spontaneous activity and stimulated responses was seen with Ca^2+^ imaging, despite increased responses to trains of electrical stimuli in nNOS^−^ neurons and increased spontaneous fast EPSPs in myenteric neurons. We considered the possibility that these may be intrinsic sensory neurons as there is evidence from guinea pig jejunum suggesting that these neurons display increased excitability following CT-exposure (Koussoulas et al., 2017). As the cell bodies of these sensory neurons tend to be larger than other functional subtypes (Qu et al., 2008), we examined whether specific subtypes of nNOS^−^ neurons may be affected by CT-exposure by comparing the cell size of responding neurons and their response amplitudes. However, no apparent differences were observed between CT and control conditions.

Myenteric neurons may contribute to the induction of CT-evoked hypersecretion, but not its maintenance, as it has been proposed that these are separate processes involving distinct neural pathways (Kordasti et al., 2006). In contrast with the hypersecretory effects that take hours to develop, CT can evoke rapid changes in motility within 15 min of exposure (Fung et al., 2010; Balasuriya et al., 2016). Thus, it is feasible that c-Fos expression results from an activation of neurons involved in these early motility effects, or even due to the distension stimuli exerted by fluid accumulation in ileal loops incubated with CT.

It is possible that Ca^2+^ imaging using GCaMP3 may not be sensitive enough to detect subtle changes in neuronal excitability. While [Ca^2+^]_i_ transients correspond well with electrical activity (Vanden Berghe et al., 2001; Michel et al., 2011; Martens et al., 2014), [Ca^2+^]_i_ signals detected using GCaMP3 may only be resolved when excitatory synaptic input initiates action potential firing (Shuttleworth and Smith, 1999; Tack and Smith, 2004). Indeed, our electrophysiological recordings of myenteric plexus preparations indicate that following CT-treatment, some myenteric neurons displayed an increase in spontaneous fast EPSPs that were revealed at a hyperpolarized membrane potential. Since stripped preparations were used, spontaneous input must originate from within the myenteric plexus and indicates that a select population of myenteric neurons is spontaneously active following CT exposure. However, the effects of CT incubation on the myenteric plexus remain unclear when considering the collective data. Nevertheless, as with VIP secretomotor neurons, the potential involvement of the myenteric plexus in the induction of CT-induced hypersecretion cannot be excluded and remain to be elucidated. As intracellular recording of submucosal neurons in mice is even more technically challenging than recording from their myenteric neurons, we did not examine the electrical properties of submucosal neurons.

Ultimately, the mouse model has both strengths and limitations, with a key advantage being the wide number of transgenic mouse models available to facilitate ENS and gastrointestinal studies. Our previous studies using the guinea pig model (Gwynne et al., 2009; Koussoulas et al., 2017) revealed prominent long lasting effects of CT on the excitability of myenteric sensory and VIP secretomotor neurons, which may better reflect the neural component of CT hypersecretion as VIP is implicated in the human condition (Bloom et al., 1976). On the other hand, the comparatively subtle neural contribution in the murine ENS found in this present study suggests that the mouse model may be more useful for studying CT effects on the mucosal epithelium. Indeed, mice have been commonly used for testing cholera therapies which target its effects on or interaction with the mucosa (Thiagarajah et al., 2004; Rivera et al., 2013). Nevertheless, the effects of CT on the ENS were only examined following pre-incubation and its more acute effects on the circuitry remain unclear. It is possible that there is a greater contribution of the ENS during the induction phase of CT hypersecretion and indeed, the mechanism by which CT increases the excitability of cholinergic submucosal neurons remain to be determined.

## Conclusion

This is the first study that combined *in vivo* and *in vitro* techniques to examine the effects of CT on both the submucosal and myenteric plexuses. It was demonstrated that CT exposure specifically induced a sustained increase in cholinergic submucosal neurons, which can include secretomotor and vasodilator neurons, and these may contribute to CT hypersecretion.

## Author contributions

AA, JB, and JF: conceived and designed the experiments; CF, KK, PU, and JF: performed the experiments; CF, KK, and JF: analyzed the data; AA, JB, and JF: contributed reagents, materials, analysis tools; CF, JB, and JF: wrote the manuscript. All authors contributed to editing and revising the manuscript. All authors read and approved the final manuscript.

### Conflict of interest statement

The authors declare that the research was conducted in the absence of any commercial or financial relationships that could be construed as a potential conflict of interest.
